# Supplementary material for: Bumblebees Learn Polarization Patterns
Source: Curr Biol. 2014 Jun 16;24(12):1415–20. doi: 10.1016/j.cub.2014.05.007 (PMC4062934; doi:10.1016/j.cub.2014.05.007)
Supplement: Document S1. Supplemental Experimental Procedures and Figures S1 and S2 [file mmc1.pdf]

Current Biology, Volume *24*

Supplemental Information

Bumblebees Learn Polarization Patterns

James J. Foster, Camilla R. Sharkey, Alicia V.A. Gaworska, Nicholas W. Roberts, Heather M. Whitney, and Julian C. Partridge

Supplemental Information

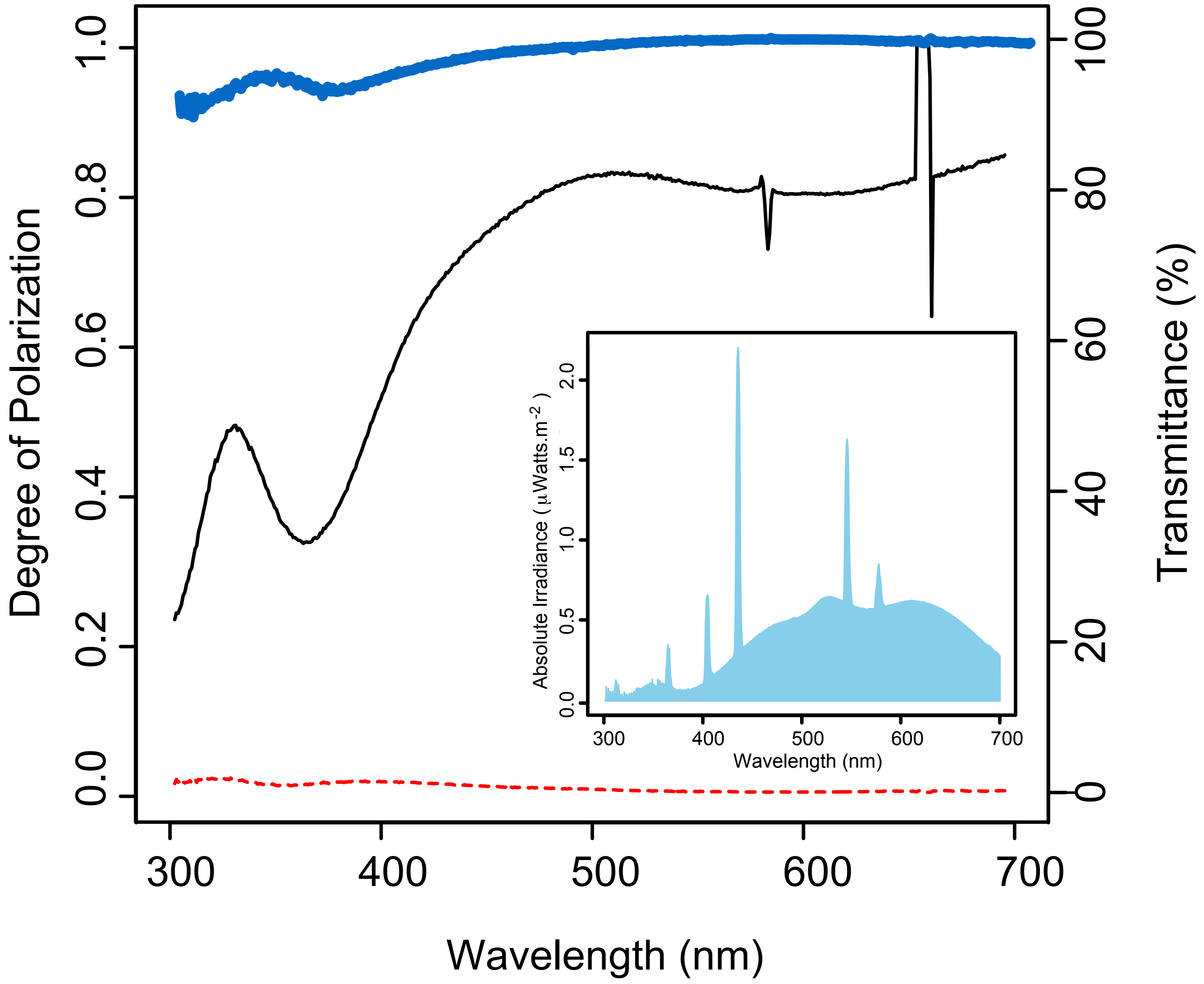

Figure S1, related to Fig.1: Transmitted Polarization across the UV-Visible Spectrum
The spectral transmission of the linear polarizers (Knight Optical HN22) used for the experiments, light polarized parallel (solid black line) and perpendicular (red dashed line) to the transmission axis of the polarizer, degree of polarization of transmitted light (blue line) and the absolute irradiance spectrum (inset: filled blue area) of illumination from the six fluorescent tubes (Sylvania Activa 172 Professional 36W), showing that the filters polarize effectively across the entire bee-visible spectrum (ca. 300–650 nm) and that UV light was available to the bees.

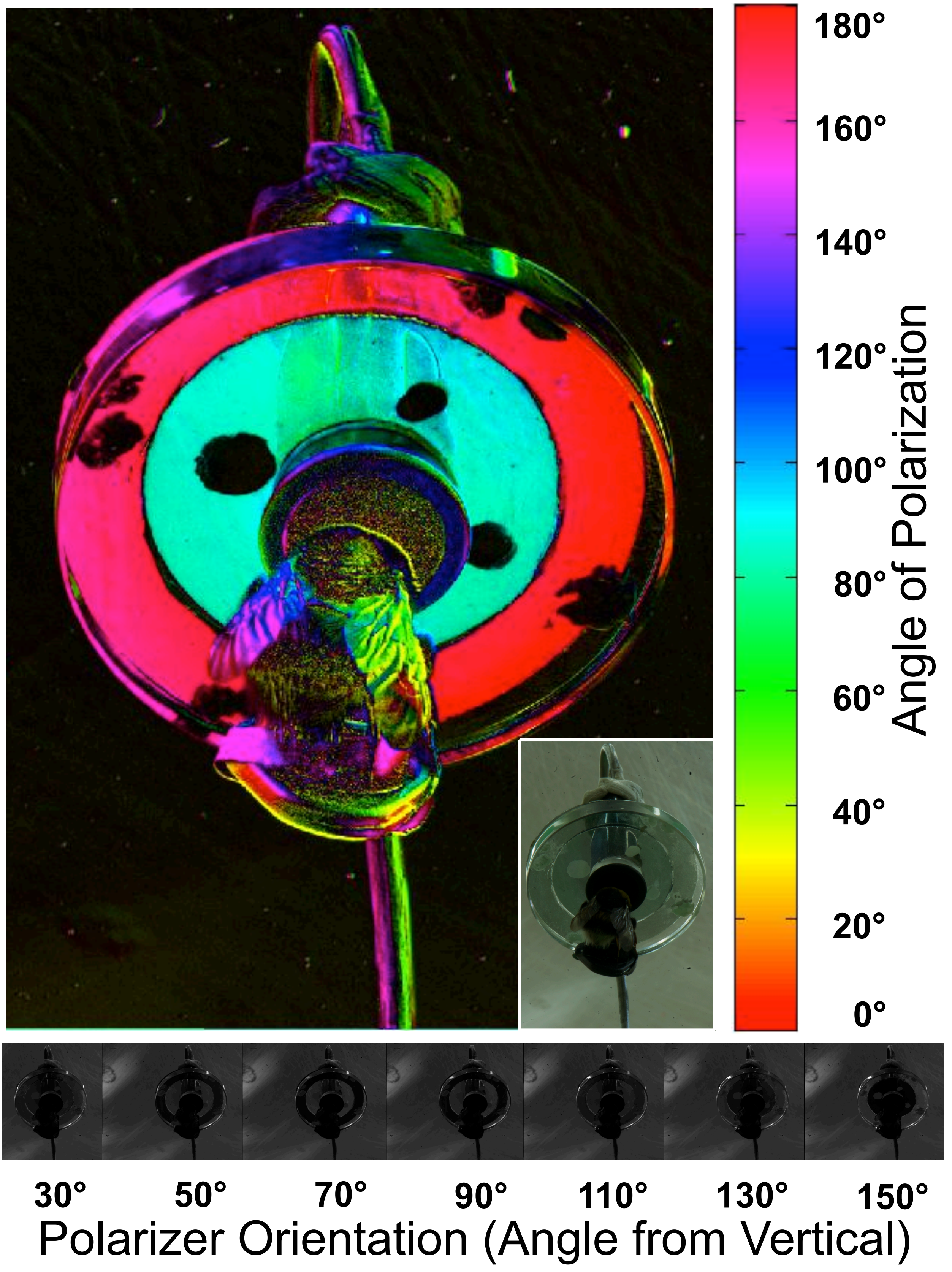

Figure S2, related to Fig.2: Bumblebee feeding from downward facing (DF) 'flower' False colour image in which colour represents angle of polarization and intensity represents degree of polarization. The colour bar represents the predominant angle of the polarization e-vector.

Supplemental Experimental Procedures

Bumblebee Colony Conditions and Flight Arena

Naïve buff-tailed bumblebees (*Bombus terrestris dalmatinus*), supplied as colonies by Syngenta-Bioline (UK) were housed in an opaque nesting box connected to a flight arena via a crawl-way tube. The flight arena (72 x 104 x 30 cm; width x length x height) was covered with UV-visible transmitting acrylic (Perspex™) sheet and the floor was covered either with green AT200 Advance Gaffa tape (Advance tapes, Thurmaston UK) to simulate foliage spectral reflection, or white card providing broad spectral reflection. Bees accessed the flight arena from their colony via the tube, with bee movements regulated by the experimenter manipulating a series of gates. Coloured Queen Marking Paints (EH Thorne (Beehives) Ltd., Wragby, UK) were used to mark, and thus identify, individual forager bees. Illumination was as described in the main text of the paper.

Differential Conditioning Experiments

Trials consisted of one hundred feeding events in which each bee was allowed to visit and sample the liquid from any of the artificial 'flowers' that were arranged in a pseudo-random grid pattern within the flight arena (Figure 1). During trials a single bee, identified previously as a motivated forager and marked with coloured queen-marking paint for identification, was allowed to forage freely within the flight arena sampling the target 'flowers'; feeding events were recorded as 'choices' between the two target types. When the foraging bee was satiated it was allowed to return to the nest to deposit the sucrose it had collected, and at this point the 'flowers' were re-arranged in a pseudo-random fashion. Once 'flowers' were rearranged the feeding reservoirs were wiped with a cotton bud soaked in 30% v/v ethanol to remove any pheromone marks left during the previous foraging bout, and target reservoirs that had been emptied by the forager were refilled. If an individual failed to return to the flight arena to continue foraging after 30 min it was not included in the final dataset, avoiding any effect of dis-habituation or memory loss. Over the course of the experiment one hundred 'choices' were recorded for each bee, with a minimum sample size of nine individuals for each experiment.
