# Supplementary material for: Bumblebees Learn Polarization Patterns
Source: Curr Biol. 2014 Jun 16;24(12):1415–20. doi: 10.1016/j.cub.2014.05.007 (PMC4062934; doi:10.1016/j.cub.2014.05.007)
Supplement: Document S2. Article plus Supplemental Information [file mmc2.pdf]

Current Biology 24, 1415–1420, June 16, 2014 ©2014 The Authors http://dx.doi.org/10.1016/j.cub.2014.05.007

Report

Bumblebees Learn Polarization Patterns

James J. Foster,[1] Camilla R. Sharkey,[1]
Alicia V.A. Gaworska,[1] Nicholas W. Roberts,[1]
Heather M. Whitney,[1] and Julian C. Partridge[1,2,*]
[1]School of Biological Sciences, University of Bristol, Woodland Road, Bristol, BS8 1UG, UK
[2]School of Animal Biology, University of Western Australia, 35 Stirling Highway, Crawley, WA 6009, Australia

Summary

Foraging insect pollinators such as bees must find and identify flowers in a complex visual environment. Bees use skylight polarization patterns for navigation [1–3], a capacity mediated by the polarization-sensitive dorsal rim area (DRA) of their eye [4, 5]. While other insects use polarization sensitivity to identify appropriate habitats [6], oviposition sites, and food sources [7], to date no nonnavigational functions of polarization vision have been identified in bees. Here we investigated the ability of bumblebees (*Bombus terrestris*) to learn polarization patterns on artificial "flowers" in order to obtain a food reward. We show that foraging bumblebees can learn to discriminate between two differently polarized targets, but only when the target artificial "flower" is viewed from below. A context for these results is provided by polarization imaging of bee-pollinated flowers, revealing the potential for polarization patterns in real flowers. Bees may therefore have the ability to use polarization vision, possibly mediated by their polarization-sensitive DRA, both for navigation and to learn polarization patterns on flowers, the latter being the first nonnavigational function for bee polarization vision to be identified.

Results and Discussion

Sensitivity to the polarization of light is a common visual ability in insects [8, 9] and other arthropods, which use it for a variety of behaviors, including sun-compass navigation [2, 4, 10], motion detection [11], detecting bodies of water [6], and determining oviposition sites [12]. Honeybees (*Apis mellifera*) were among the first animals to be identified as being polarization sensitive [1] and to use the polarization-sensitive dorsal rim area (DRA) of their eye to identify the position of the sun for sun-compass navigation [2, 4, 10]. The role of polarization sensitivity in insect foraging, however, is far less well known, although the butterfly *Papilio xuthus* can learn to associate certain angles of polarization with food [7] (albeit confounding polarization with color), and polarization vision has not hitherto been suggested in the context of bee foraging. Both pollinator and plant fitness are greatly dependent on the ability of pollinators to discriminate flowers accurately, and bees have been shown to be able to use a wide range of floral cues, including color, shape, texture, volatiles, and temperature, to improve identification and recognition of flowers [13, 14]. Recent findings have added floral humidity and electric fields as additional modalities with which pollinators can discriminate flowers [15, 16], and it is advantageous for a plant to produce a multimodal array of signals that a pollinator can utilize effectively [17]. Could polarization sensitivity therefore function as an additional floral cue for bees?

Orientation of Artificial Flowers Affects Learning Ability for Polarization Patterns

We investigated whether bumblebees could learn polarization patterns of artificial "flowers" and found that their ability to distinguish between two differently polarized targets was dependent on viewing direction. Freely foraging bumblebees flying in an experimental arena learned to differentiate between rewarding (sucrose solution providing) and aversive (quinine solution providing) artificial "flowers" (Figure 1) in a single learning session. Patterns of polarization (Figure 2) were either rewarding, type "contrast" (in which the two linearly polarized components of the bull's-eye-patterned target were oriented at 90° to each other), or aversive, type "plain" (having the polarized components oriented in the same direction). In one treatment "flowers" were oriented to be upward facing (UF), such that they were viewed by the ventral region of the bee's eye, and in another treatment "flowers" were downward facing (DF), such that they were viewed by the dorsal part of the bee's eye, including the polarization-sensitive DRA. For DF "flowers" in two experiments (DF1 and DF2), nested mixed-effects models of learning for repeated experiments provided significantly better fits than null models (DF1: Δdeviance = 13.142, degrees of freedom [df] = 1, $p < 0.01$; DF2: Δdeviance = 21.761, df = 1, $p < 0.01$) and had lower Akaike Information Criterion (AIC) values (DF1: AIC 923.25 versus 912.11; DF2: AIC 1288.8 versus 1,269.1), indicating that foraging experience led to a greater proportion of rewarding "flowers" being chosen (Figure 3). For UF "flowers," this was not the case in two experiments, UF1 and UF2 (UF1: AIC 1,251.3 versus 1,252.4, Δdeviance = 0.8733, df = 1, p = 0.35; UF2: AIC 1,390.3 versus 1,392.3, Δdeviance = 0.0137, df = 1, p = 0.9067), and the task was not learned (Figure 3). The number of trials required to reach a maximum proportion of correct responses (100 trials) is longer than that required by bees to learn most colors and shapes in choice experiments [18–21], indicating that bees find this task difficult (this number of trials is equivalent to that needed for bees to learn a "very high" color similarity [19]). In nature, however, polarization patterns may be learned more easily in concert with other cues as part of a multicomponent signal [22, 23].

Many insect species possess polarization sensitive areas in the ventral and lateral regions of their eyes [6, 24], or indeed throughout their eye [7, 12], in addition to the DRA. In honeybees, polarization sensitivity of the compound eyes tends to be limited to the DRA [2, 4, 10] and to be UV mediated. Nevertheless, ommatidia in the DRA of honeybees express both UV- and green-sensitive visual pigments [25], and the DRA photoreceptors of bumblebees express more than one UV pigment [26], potentially broadening spectral sensitivity. In addition, there is electrophysiological evidence that bumblebees have both blue and UV polarization sensitivity in their main retina [27], as well as polarization-sensitive ocelli [28]. Consequently, we used light sources that included the full bee-visible spectrum, including the UV, and employed linear polarizers that polarized effectively across the whole of this spectrum (Figure S1 available online). In the color-sensitive

*Correspondence: j.c.partridge@bristol.ac.uk
This is an open access article under the CC BY license (http://creativecommons.org/licenses/by/3.0/).

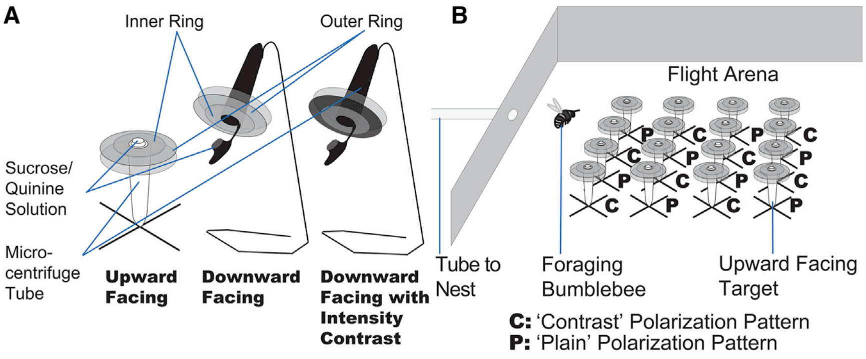

Figure 1. Experimental Setup for Differential Conditioning Experiments

(A) Artificial "flower" targets. Each target consisted of a microcentrifuge tube and a ring of 5-mm-thick UV-visible transparent acrylic sheet (Perspex) on which were mounted inner and outer rings of linear polarizer. The targets for the "downward-facing with intensity contrast" (DFIC) treatment were identical to those in the "downward-facing" experiment, except that an additional layer of neutral density filter was attached to each ring.

(B) Setup in the experimental arena. An even distribution of different target types was maintained for each experiment, and individual targets were pseudorandomly shuffled between foraging bouts, between which the bee returned to the nest to deposit sucrose.

See also Figure S1.

regions of the bee's compound eye, adaptations such as twisted rhabdoms [10] reduce polarization sensitivity to avoid confounding color and polarization [29], as occurs in the butterfly *Papilio* [7, 12], which lacks twisted rhabdoms. Since bumblebees were trained successfully to distinguish between two polarization patterns, but only when presented dorsally (DF "flowers"), our data suggest that flower discrimination can be mediated by polarization sensitivity, but only when the flowers are viewed by the dorsal region of the eye. A further requirement may be that the pattern is simple and spatially course as the spatial resolution of the DRA is low in most polarization sensitive insects [5], including bumblebees [30], although we cannot eliminate the possibility that they could detect "finer" polarization patterns with higher spatial frequencies than we presented. The results of previous attempts to demonstrate polarization sensitivity behaviorally in *Bombus* spp. have been somewhat ambiguous and related to polarotaxis rather than target identification in foraging (e.g., [31]), and this study represents the first clear evidence that bumblebees may be trained to polarization patterns.

Learning of Polarization Patterns Is Independent of Intensity Cues

In a further experiment, bees were again presented with downward-facing "flowers" with a polarization pattern that indicated whether the "flower" was rewarding or aversive, but with an added intensity contrast pattern that was unrelated to the rewarding status of the "flower" (downward facing with intensity contrast, DFIC), such that there were four types of "flower": condition 1, polarization contrast with darker outer ring (rewarding); condition 2, polarization contrast with lighter outer ring (rewarding); condition 3, polarization plain with darker outer ring (aversive); and condition 4, polarization plain with lighter outer ring (aversive). Nested mixed-effects models of learning effects again provided significantly better fits than null models and had lower AIC values (AIC 1,431.8 versus 1,426.6; Δdeviance = 7.2162, df = 1, $p < 0.01$). Not only were the bumblebees capable of learning to differentiate between the two polarization patterns, but intensity contrast type (darker outer or inner ring) did not affect the choice of polarization pattern (AIC 4,817.9 versus 4,819.6; Δdeviance = 0.3283, df = 1, p = 0.5667), indicating that they can learn to ignore intensity patterns in favor of polarization patterns when these patterns are visible to the frontodorsal region of the eye, including the DRA. This result suggests that the polarization cues are not simply interpreted as patterns of intensity contrasts by the bees.

Polarimetry of Downward-Facing Flowers

Our results suggest that bees may be able to learn polarization patterns on downward-facing, pendant, natural flowers. The orientation of pendant flowers will impart directionality to pollinators as they approach [32], and it has been estimated that 53% of flower species do not face upward [33]. The occurrence of polarization patterns presented in such a way as to be visually accessible to the dorsofrontal region of the eye, including the bees' DRA, could therefore be widespread, particularly as various mechanisms such as internal reflections

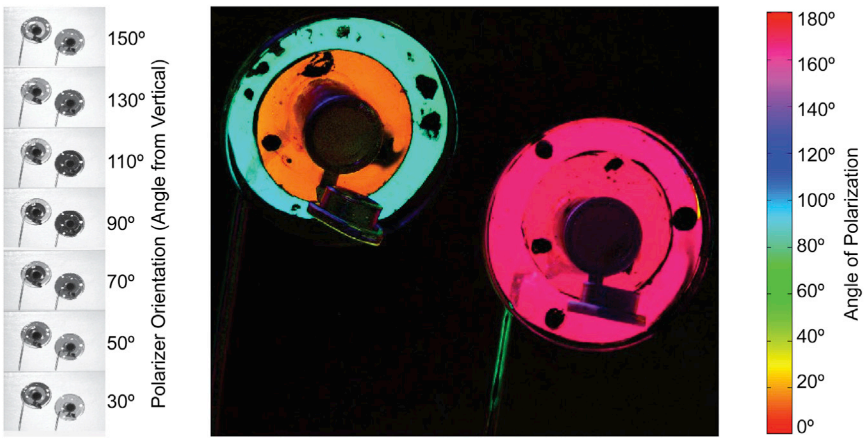

Figure 2. False-Color Image of Downward-Facing Polarized Targets

Left: images taken through different camera filter linear polarizer orientations and used to calculate the angle and degree of polarization for each pixel.

Center: false-color image of the two target polarization patterns calculated from images in the left panel. Targets, of the two different types used in this study, are shown scaled by the angle of polarization (color) and degree of polarization (brightness). For the "contrast" target (left image), the inner and outer rings have perpendicular polarizer orientations, whereas for the "plain" target (right image), both rings are oriented in the same direction.

Right: color scale for the false-color image showing the angle of polarization.

See also Figure S2.

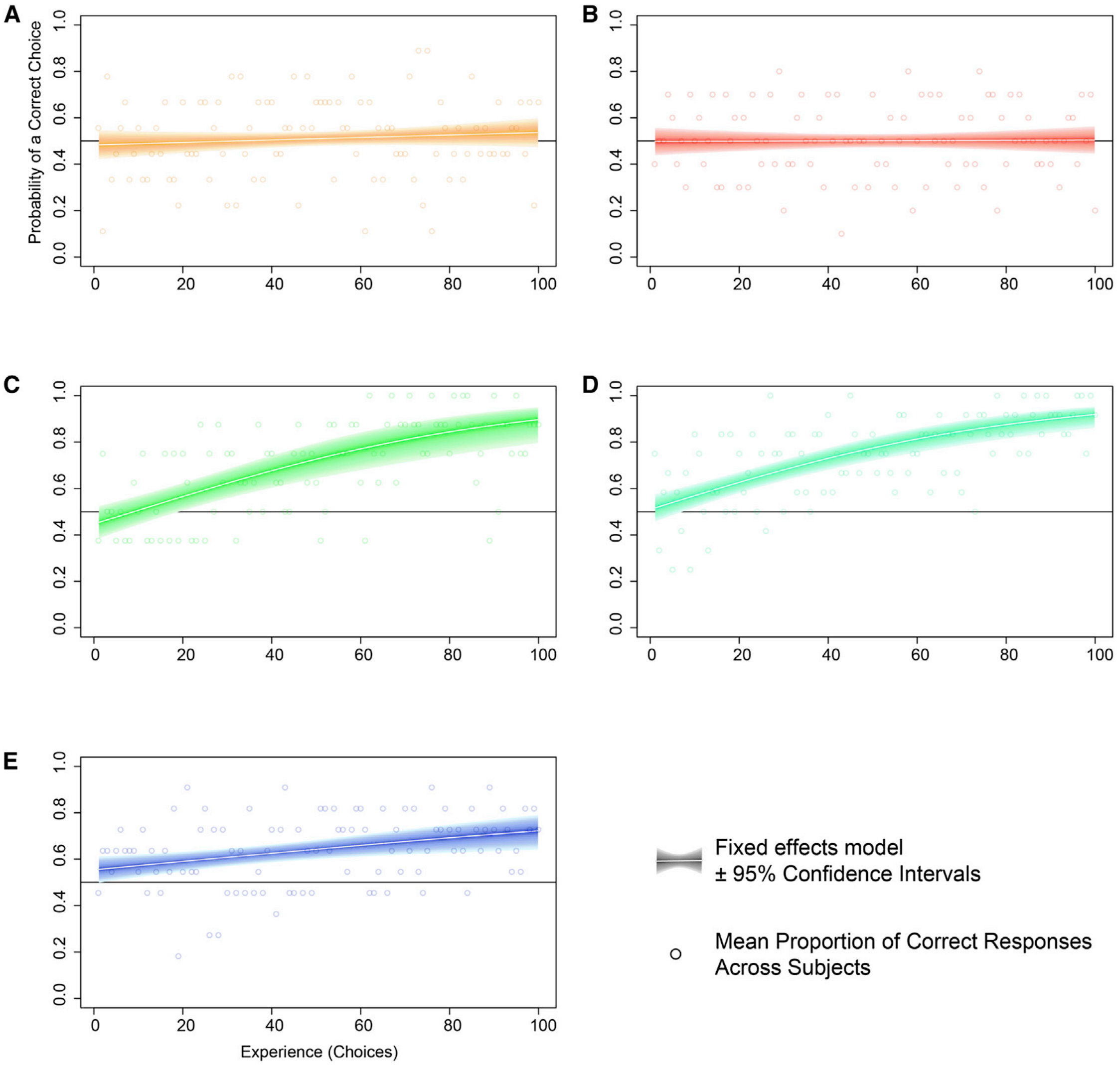

Figure 3. Fitted Learning Curves for Each Experiment

Mixed-effects models indicate learning only in the case of downward-facing (DF) "flowers" (C and D; experimental blocks 1 and 2, respectively), with lower rates of learning for the DFIC treatment (E) in which polarization cues were mixed with brightness cues. Bees could not learn polarization cues from upward-facing (UF) "flowers" (A and B; experimental blocks 1 and 2, respectively). Graphs show means ± 95% confidence limits in all cases. An effect of experience on success rate indicates the acquisition of useful information over time, so it can be concluded that in treatments DF1, DF2, and DFIC, the animals were able to learn the differences in polarization pattern between the rewarding and aversive targets, whereas in treatments UF1 and UF2 they were not.

[34] and specular reflection [35] have the potential to produce polarization patterns in petals. Petals in particular have a range of epidermal morphologies, at both macro and micro scale, that could contribute to polarization signals [36]. These occur in patterns [36, 37], and these patterns of petal surface structure were found to correspond to patterns of polarization in three phylogenetically distinct species (the gentian *Eustoma russellianum*, family Gentianaceae; the garden anemone *Anemone coronaria*, family Ranunculaceae; and the tree mallow *Lavatera* × *clementii* "Rosea", family Malaceae; Figure 4). Since these patterns in petal surface structure have previously been described in several other floral species (for example, flower-of-an-hour *Hibiscus trionum*, family Malvacea; Chilean bellflower *Nolana paradoxa*, family Solanaceae; the tulip *Tulipa humilis*, family Liliaceae; and the meadow vetchling *Lathyrus pratensis*, family Fabaceae) and are thought to be widespread in multimodal flowers [36, 37], the corresponding patterns in polarization are likely to be equally prevalent. Basic image processing to calculate angle of polarization, as presented here and elsewhere (e.g., [8] and references therein), can provide valuable information about surface, orientation, and curvature. Bumblebees are known to take flower orientation into account when foraging as prior understanding of flower orientation affects flower handling [38], the minimization of handling time enhancing foraging efficiency. In addition, light reflected from downward-facing flowers has the potential to contrast with skylight polarization patterns, potentially facilitating bees' detection and identification of flowers.

Conclusions

In addition to the well-established use of polarization sensitivity for sun-compass navigation, we show that bumblebees are able to learn to identify polarization patterns associated with a food source when foraging. Visual polarization information is interpreted independently from intensity information as,

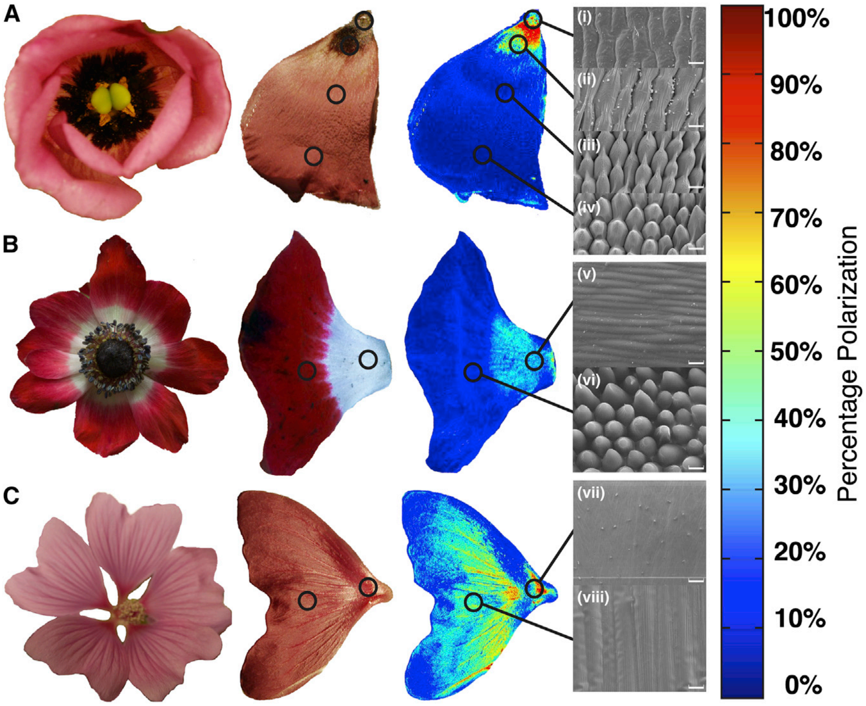

Figure 4. Flower Polarization Patterns Are Associated with Surface Structures

Color photographs of whole flowers and individual petals (first and second columns, respectively) taken under different artificial light sources and percentage polarization false-color images of fresh petals (third column) of the gentian *Eustomia russellianum* (A), the garden anemone *Anemone coronaria* (B), and the tree mallow *Lavatera* × *clementii* "Rosea" (C). Scanning electron microscopy images, labeled (i) to (viii), of resin casts of the petals (fourth column) show the petal surface structures that result in the differently polarized regions (scale bars, 20 μm). The color bar (right margin) shows the percentage polarization of the false-color images.

when only polarization indicates profitability, intensity patterns are ignored. While this capacity is apparently restricted to the frontodorsal field of view in bumblebees (and so possibly mediated by the DRA), bee-pollinated pendant (downward-facing) flowers represent a high proportion of angiosperm species. Our polarimetric imaging of flowers (Figure 4) indicates that petals produce polarized reflections due to their surface cellular organization and that these patterns are presented in such a way as to be available to pollinators approaching flowers from the below. Flower polarization patterns may therefore provide important information about flower shape and petal orientation that can be learned by bees, as well as providing cues for the discrimination of rewarding and nonrewarding food sources. Floral polarization patterns may therefore serve as signals to pollinators and rank alongside color and intensity patterns as foraging cues, extending the use of polarization vision by bumblebees from purely navigational purposes to the critical assessment of salient objects in their multisensory landscape.

Experimental Procedures

Artificial Flower Design

Artificial "flower" targets were constructed from black microcentrifuge tubes (10 mm mouth diameter, 2 ml volume) on top of which were mounted 38 mm diameter rings of 5-mm-thick UV-visible transmitting Perspex acrylic sheet. Two concentric rings of linear polarizing filter (HN22; Knight Optical), with 38 and 24 mm outer diameters, were affixed to the underside of the Perspex (Figure 1). For UF "flowers", the acrylic surface was horizontal, facing upward, and the upturned lid of a microcentrifuge tube acted as a feeding reservoir for foraging bees; for the DF targets, the acrylic surface was 0°–30° from horizontal, facing downward, and the lid of the microcentrifuge tube was left attached and open. Between trials, bees were allowed to feed from similar "flowers" in which rings of neutral-density filter replaced the polarizing filter. Two types of target, arranged in pseudorandom grids, were used for differential conditioning: contrast targets with polarizer rings that transmitted perpendicular polarization angles, and plain targets with parallel-oriented polarizer rings (Figure 2). The feeding reservoir on each target contained either a 30% w/v sucrose solution, on the contrast targets, or 0.12% w/v quinine hemisulphate hydrate ($C_{20}H_{24}N_2O_2 \cdot 0.5H_2O_4S \cdot H_2O$) solution, on the plain targets. The former acted as a reward, and the latter as a distasteful deterrent to foraging from these targets, differential conditioning having been shown to be more effective in honeybees than reward alone [19, 21, 39].

Downward-Facing "Flowers" with Intensity Contrasts

Targets contrasting in intensity and polarization were constructed by addition of a further two concentric rings (with the same diameters as the polarization filters) of 0.3 and 0.9 neutral-density filters (209 and 211 Lee Filters) on top of the polarizing filters (Figure 1). These targets were of the four different conditions described above (see the Results and Discussion). Four "flowers" of each condition were used in each trial and were arranged in a four-by-four Latin square. So that the effect of spatial learning of the target positions could be reduced, when the forager returned to the nest between foraging bouts, targets were rearranged such that (1) no target was in its previous place, (2) no place was occupied by a target of the same condition as previously, and (3) the four different conditions all formed a Latin square.

Differential Conditioning Experiments

The procedure for differential conditioning in *B. terrestris* was based on methods used to great effect in studies of sensitivity to color, surface microstructure, and electric field strength [16, 18, 38]. One hundred choices were recorded for each individual as the choice of either a rewarded or aversive target for a minimum of nine motivated foragers per treatment. All experiments were conducted under the guidelines and rules laid down by the University of Bristol. Further details of the differential conditioning procedure can be found in the Supplemental Experimental Procedures.

Bumblebee Colony Conditions and Flight Arena

Animal husbandry methods are detailed in the Supplemental Experimental Procedures. Naive *B. terrestris* colonies were supplied by Syngenta Bioline. The flight arena (72 cm wide × 104 cm long × 30 cm high) was covered with UV-visible transmitting acrylic (Perspex) sheet. All work was conducted under the University of Bristol guidelines for animal experiments.

Lights and Light Levels

Illumination for behavioral experiments was provided by six Sylvania Activa 172 Professional 36 W fluorescent tubes powered by Phillips high-frequency ballasts to have a flicker frequency greater than 1,200 Hz and run on a 12:12 hr daily light:dark regime. Light levels in the flight arena were measured using a Hanastech Quantitherm Lightmeter and averaged 20.3 μmol/m^2/s (SEM 0.214), and the spectrum was measured with a USB2000 Spectrophotometer (Ocean Optics), P600-10-UV/VIS optical fiber (Ocean Optics), and a CC-3-UV-T cosine-corrected irradiance probe (Ocean Optics). Illumination encompassed the whole bee-visible spectrum, including the UV (Figure S1).

Statistical Analysis

Insect learning curves associated with each treatment were obtained by pooling of data from all individuals. Since there were strong between-subject differences in both ability to learn and rate of acquisition of learning, logistic mixed-effects models were chosen to account for random between-subject effects. Logistic regression models, as employed here, are commonly used to fit relationships between binomial response data and factors such as experience and condition [17, 23, 40] and may be used as a link function for fitting generalized linear mixed-effects models [41, 42]. For the DFIC treatment, a logistic mixed-effects model was also fitted for the effect of the interaction of intensity contrast type with experience (i.e., number of choices) on the proportion of correct responses, to investigate whether intensity contrasts contributed to learning. All nested models were compared with a simpler random-effects model using the change in deviance on removal of a term from the model, as well as the Akaike Information Criterion [43], to test for each model's ability to describe the data. Logistic mixed-effects models were fitted with the lme4 package (version 1.0-5) in R 3.0.2 [41].

Polarization Imaging

Color-coded images showing the degree of polarization and angle of polarization for each pixel were calculated from digital photographs of artificial "flowers" and natural bee-pollinated flowers provided by the University of Bristol Botanic Gardens. Images were recorded with a Nikon D70 DSLR and stored as NEF (RAW) files. The camera was mounted on a heavy tripod, and a subject flower was held immobile in a darkroom laboratory under controlled, directional, invariant illumination provided from a fiber optic illuminator (Schott KL1500 Electronic Light Source). A sequence of eight images of each flower was recorded: seven with a linear polarizing filter rotated in 20° increments and a final "dark" image recorded with a lens cap in place. RAW images were transferred to computer and converted to uncompressed TIFF files using open-source RAW image-decoding software (DCRAW, [44]), maintaining pixel bit number linearity with pixel exposure, and keeping the four Bayer-masked CCD channels separate. These RGGB color channels were separated using a MATLAB (version 8.1.0.604; MathWorks) program and saved as uncompressed TIFF files, with one color channel being selected for further analysis. The corresponding TIFF images were aligned using ImageJ [45] with the Turboreg and Stackreg plugins [46]. A further MATLAB program was used to calculate the Stokes parameters for each pixel across the series of aligned images and produced images representing, variously, predominant angle of polarization, degree of polarization, and predominant angle of polarization with pixel brightness weighted by the degree of polarization, displayed as a color-coded image (Figures 2, 4, and S2).

Supplemental Information

Supplemental Information includes Supplemental Experimental Procedures and two figures and can be found with this article online at http://dx.doi.org/10.1016/j.cub.2014.05.007.

Acknowledgments

We thank I. Cuthill for statistical advice and R. Woodfield, W. Amos, and J. Ruščić for assistance with experiments. We are grateful for funding from BBSRC grants BB/H01635X/1 (to N.W.R. and J.C.P.) and BB/G022917/1 (to N.W.R.) and from ERC project number 260920 (to H.M.W.).

Received: January 9, 2014
Revised: March 19, 2014
Accepted: May 2, 2014
Published: June 5, 2014

References

1. von Frisch, K. (1948). Die Polarisation des Himmelslichtes als orientierender Faktor bei den Tänzen der Bienen. Experientia *5*, 142–148.
2. Rossel, S., Wehner, R., and Lindauer, M. (1978). E-vector orientation in bees. J. Comp. Physiol. A Neuroethol. Sens. Neural Behav. Physiol. *125*, 1–12.
3. Kraft, P., Evangelista, C., Dacke, M., Labhart, T., and Srinivasan, M.V. (2011). Honeybee navigation: following routes using polarized-light cues. Philos. Trans. R. Soc. Lond. B Biol. Sci. *366*, 703–708.
4. Menzel, R., and Snyder, A. (1974). Polarised light detection in the bee, Apis mellifera. J. Comp. Physiol. A Neuroethol. Sens. Neural Behav. Physiol. *88*, 247–270.
5. Labhart, T., and Meyer, E.P. (1999). Detectors for polarized skylight in insects: a survey of ommatidial specializations in the dorsal rim area of the compound eye. Microsc. Res. Tech. *47*, 368–379.
6. Horváth, G., and Kriska, G. (2008). Polarization vision in aquatic insects and ecological traps for polarotactic insects. In Aquatic Insects: Challenges to Populations, J. Lancaster and R.A. Briers, eds. (Wallingford: CABI), pp. 204–229.
7. Kelber, A., Thunell, C., and Arikawa, K. (2001). Polarisation-dependent colour vision in Papilio butterflies. J. Exp. Biol. *204*, 2469–2480.
8. Horváth, G., and Varjú, D. (2004). Polarized Light in Animal Vision (Heidelberg: Springer-Verlag).
9. Roberts, N.W., Porter, M.L., and Cronin, T.W. (2011). The molecular basis of mechanisms underlying polarization vision. Philos. Trans. R. Soc. Lond. B Biol. Sci. *366*, 627–637.
10. Wehner, R., Bernard, G.D., and Geiger, E. (1975). Twisted and non-twisted rhabdoms and their significance for polarization detection in the bee. J. Comp. Physiol. A Neuroethol. Sens. Neural Behav. Physiol. *104*, 225–245.
11. Glantz, R.M., and Schroeter, J.P. (2006). Polarization contrast and motion detection. J. Comp. Physiol. A Neuroethol. Sens. Neural Behav. Physiol. *192*, 905–914.
12. Kelber, A. (1999). Why 'false' colours are seen by butterflies. Nature *402*, 251.
13. Raguso, R.A. (2004). Flowers as sensory billboards: progress towards an integrated understanding of floral advertisement. Curr. Opin. Plant Biol. *7*, 434–440.
14. Leonard, A.S., and Papaj, D.R. (2011). 'X' marks the spot: the possible benefits of nectar guides to bees and plants. Funct. Ecol. *25*, 1–9.
15. von Arx, M., Goyret, J., Davidowitz, G., and Raguso, R.A. (2012). Floral humidity as a reliable sensory cue for profitability assessment by nectar-foraging hawkmoths. Proc. Natl. Acad. Sci. USA *109*, 9471–9476.
16. Clarke, D., Whitney, H., Sutton, G., and Robert, D. (2013). Detection and learning of floral electric fields by bumblebees. Science *340*, 66–69.
17. Kaczorowski, R.L., Leonard, A.S., Dornhaus, A., and Papaj, D.R. (2012). Floral signal complexity as a possible adaptation to environmental variability: a test using nectar-foraging bumblebees, Bombus impatiens. Anim. Behav. *83*, 905–913.
18. Dyer, A.G., and Chittka, L. (2004a). Fine colour discrimination requires differential conditioning in bumblebees. Naturwissenschaften *91*, 224–227.
19. Dyer, A.G., and Chittka, L. (2004b). Bumblebees (Bombus terrestris) sacrifice foraging speed to solve difficult colour discrimination tasks. J. Comp. Physiol. A Neuroethol. Sens. Neural Behav. Physiol. *190*, 759–763.
20. Giurfa, M. (2004). Conditioning procedure and color discrimination in the honeybee Apis mellifera. Naturwissenschaften *91*, 228–231.
21. Avarguès-Weber, A., de Brito Sanchez, M.G., Giurfa, M., and Dyer, A.G. (2010). Aversive reinforcement improves visual discrimination learning in free-flying honeybees. PLoS ONE *5*, e15370.
22. Rowe, C. (1999). Receiver psychology and the evolution of multicomponent signals. Anim. Behav. *58*, 921–931.
23. Kulahci, I.G., Dornhaus, A., and Papaj, D.R. (2008). Multimodal signals enhance decision making in foraging bumble-bees. Proc. Biol. Sci. *275*, 797–802.
24. Wernet, M.F., Velez, M.M., Clark, D.A., Baumann-Klausener, F., Brown, J.R., Klovstad, M., Labhart, T., and Clandinin, T.R. (2012). Genetic dissection reveals two separate retinal substrates for polarization vision in Drosophila. Curr. Biol. *22*, 12–20.
25. Wakakuwa, M., Kurasawa, M., Giurfa, M., and Arikawa, K. (2005). Spectral heterogeneity of honeybee ommatidia. Naturwissenschaften *92*, 464–467.
26. Spaethe, J., and Briscoe, A.D. (2005). Molecular characterization and expression of the UV opsin in bumblebees: three ommatidial subtypes in the retina and a new photoreceptor organ in the lamina. J. Exp. Biol. *208*, 2347–2361.
27. Meyer-Rochow, V.B. (1981). Electrophysiology and histology of the eye of the bumblebee Bombus hortorum (L.) (Hymenoptera: Apidae). J. R. Soc. N. Z. *11*, 123–153.
28. Ribi, W., Warrant, E., and Zeil, J. (2011). The organization of honeybee ocelli: regional specializations and rhabdom arrangements. Arthropod Struct. Dev. *40*, 509–520.

29. Wehner, R., and Bernard, G.D. (1993). Photoreceptor twist: a solution to the false-color problem. Proc. Natl. Acad. Sci. USA *90*, 4132–4135.
30. Macuda, T., Gegear, R.J., Laverty, T.M., and Timney, B. (2001). Behavioural assessment of visual acuity in bumblebees (Bombus impatiens). J. Exp. Biol. *204*, 559–564.
31. Jacobs-Jessen, U.F. (1959). Zur Orientierung der Hummeln und Einiger Anderer Hymenopteren. J. Comp. Physiol. A Neuroethol. Sens. Neural Behav. Physiol. *41*, 597–641.
32. Fenster, C.B., Armbruster, W.S., and Dudash, M.R. (2009). Specialization of flowers: is floral orientation an overlooked first step? New Phytol. *183*, 502–506.
33. Rands, S.A., Glover, B.J., and Whitney, H.M. (2011). Floral epidermal structure and flower orientation: getting to grips with awkward flowers. Arthropod-Plant Interact. *5*, 279–285.
34. Kay, Q., Daoud, H., and Stirton, C. (1981). Pigment distribution, light reflection and cell structure in petals. Bot. J. Linn. Soc. *83*, 57–84.
35. Horváth, G., Gál, J., Labhart, T., and Wehner, R. (2002). Does reflection polarization by plants influence colour perception in insects? Polarimetric measurements applied to a polarization-sensitive model retina of Papilio butterflies. J. Exp. Biol. *205*, 3281–3298.
36. Whitney, H.M.H.M., Kolle, M., Andrew, P., Chittka, L., Steiner, U., and Glover, B.J. (2009b). Floral iridescence, produced by diffractive optics, acts as a cue for animal pollinators. Science *323*, 130–133.
37. Whitney, H.M., Kolle, M., Alvarez-Fernandez, R., Steiner, U., and Glover, B.J. (2009c). Contributions of iridescence to floral patterning. Commun. Integr. Biol. *2*, 230–232.
38. Whitney, H.M., Chittka, L., Bruce, T.J.A., and Glover, B.J. (2009a). Conical epidermal cells allow bees to grip flowers and increase foraging efficiency. Curr. Biol. *19*, 948–953.
39. Chittka, L., Dyer, A.G., Bock, F., and Dornhaus, A. (2003). Psychophysics: bees trade off foraging speed for accuracy. Nature *424*, 388.
40. Hartz, S.M., Ben-Shahar, Y., and Tyler, M. (2001). Logistic growth curve analysis in associative learning data. Anim. Cogn. *3*, 185–189.
41. Bolker, B.M. (2013). Linear Mixed-Effects Models Using Eigen and S4. https://github.com/lme4/lme4/.
42. Bates, D. (2011). Linear Mixed Model Implementation in lme4. http://www.icesi.edu.co/CRAN/web/packages/lme4/vignettes/Implementation.pdf.
43. Akaike, H. (1974). A new look at the statistical model identification. IEEE Trans. Automat. Contr. *19*, 716–723.
44. Coffin, D. (2013) Decoding Raw Digital Images in Linux. http://www.cybercom.net/~dcoffin/dcraw/
45. Schneider, C.A., Rasband, W.S., and Eliceiri, K.W. (2012). NIH Image to ImageJ: 25 years of image analysis. Nat. Methods *9*, 671–675.
46. Thévenaz, P., Ruttimann, U.E., and Unser, M. (1998). A pyramid approach to subpixel registration based on intensity. IEEE Trans. Image Process. *7*, 27–41.

Current Biology, Volume *24*

Supplemental Information

Bumblebees Learn Polarization Patterns

James J. Foster, Camilla R. Sharkey, Alicia V.A. Gaworska, Nicholas W. Roberts, Heather M. Whitney, and Julian C. Partridge

Supplemental Information

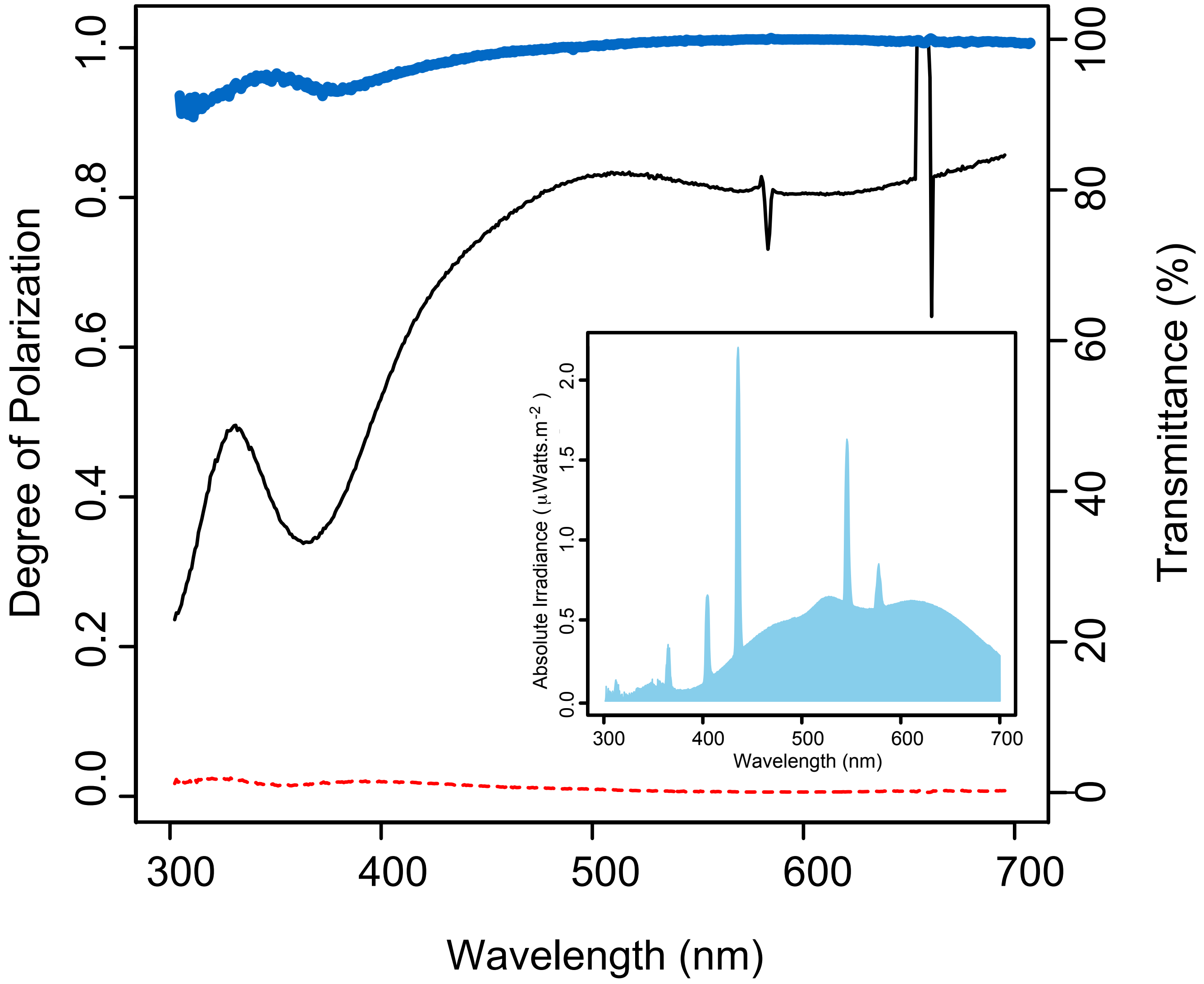

Figure S1, related to Fig.1: Transmitted Polarization across the UV-Visible Spectrum
The spectral transmission of the linear polarizers (Knight Optical HN22) used for the experiments, light polarized parallel (solid black line) and perpendicular (red dashed line) to the transmission axis of the polarizer, degree of polarization of transmitted light (blue line) and the absolute irradiance spectrum (inset: filled blue area) of illumination from the six fluorescent tubes (Sylvania Activa 172 Professional 36W), showing that the filters polarize effectively across the entire bee-visible spectrum (ca. 300–650 nm) and that UV light was available to the bees.

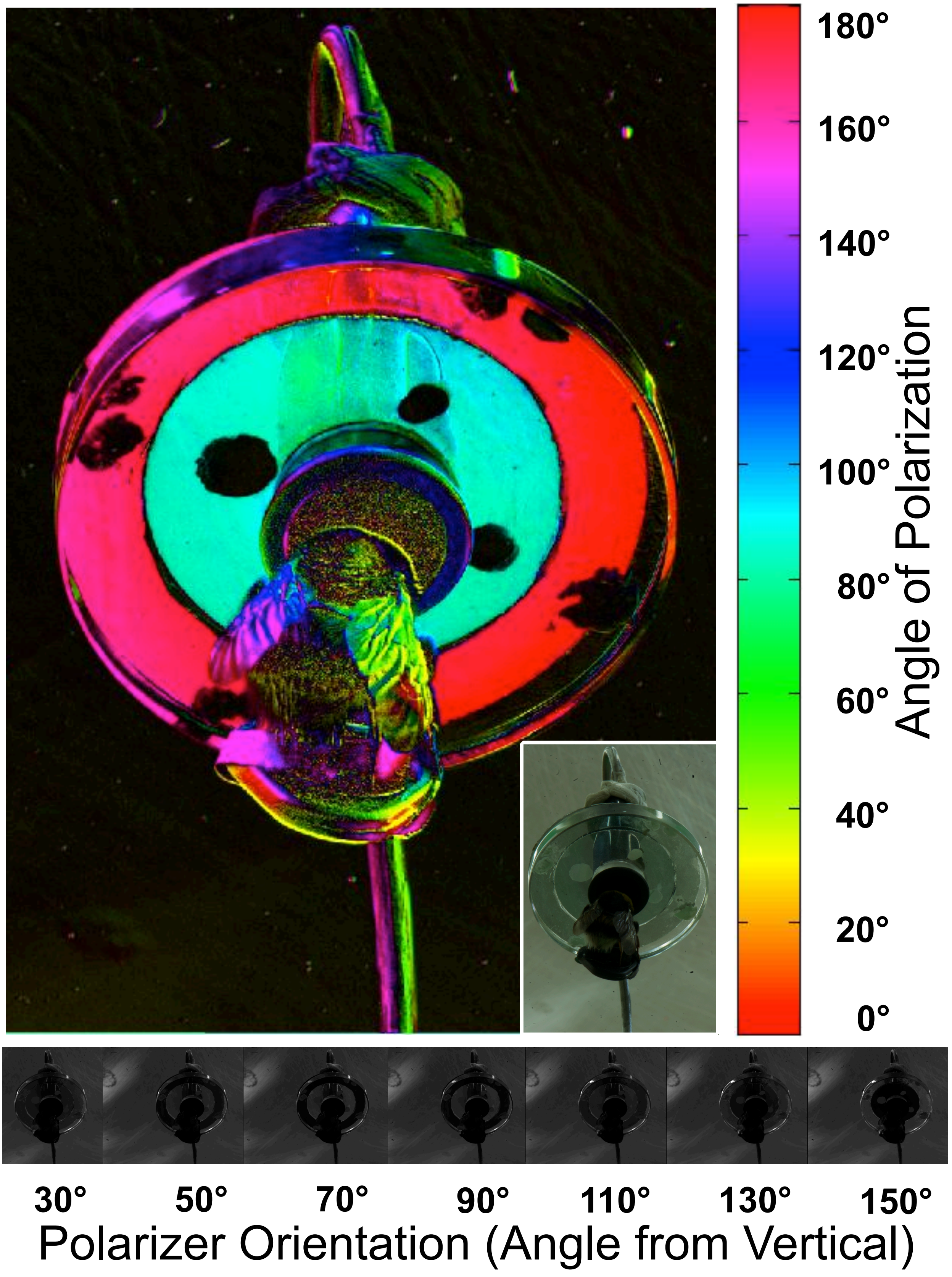

Figure S2, related to Fig.2: Bumblebee feeding from downward facing (DF) 'flower' False colour image in which colour represents angle of polarization and intensity represents degree of polarization. The colour bar represents the predominant angle of the polarization e-vector.

Supplemental Experimental Procedures

Bumblebee Colony Conditions and Flight Arena

Naïve buff-tailed bumblebees (*Bombus terrestris dalmatinus*), supplied as colonies by Syngenta-Bioline (UK) were housed in an opaque nesting box connected to a flight arena via a crawl-way tube. The flight arena (72 x 104 x 30 cm; width x length x height) was covered with UV-visible transmitting acrylic (Perspex™) sheet and the floor was covered either with green AT200 Advance Gaffa tape (Advance tapes, Thurmaston UK) to simulate foliage spectral reflection, or white card providing broad spectral reflection. Bees accessed the flight arena from their colony via the tube, with bee movements regulated by the experimenter manipulating a series of gates. Coloured Queen Marking Paints (EH Thorne (Beehives) Ltd., Wragby, UK) were used to mark, and thus identify, individual forager bees. Illumination was as described in the main text of the paper.

Differential Conditioning Experiments

Trials consisted of one hundred feeding events in which each bee was allowed to visit and sample the liquid from any of the artificial ‘flowers’ that were arranged in a pseudo-random grid pattern within the flight arena (Figure 1). During trials a single bee, identified previously as a motivated forager and marked with coloured queen-marking paint for identification, was allowed to forage freely within the flight arena sampling the target ‘flowers’; feeding events were recorded as ‘choices’ between the two target types. When the foraging bee was satiated it was allowed to return to the nest to deposit the sucrose it had collected, and at this point the ‘flowers’ were re-arranged in a pseudo-random fashion. Once ‘flowers’ were rearranged the feeding reservoirs were wiped with a cotton bud soaked in 30% v/v ethanol to remove any pheromone marks left during the previous foraging bout, and target reservoirs that had been emptied by the forager were refilled. If an individual failed to return to the flight arena to continue foraging after 30 min it was not included in the final dataset, avoiding any effect of dis-habituation or memory loss. Over the course of the experiment one hundred ‘choices’ were recorded for each bee, with a minimum sample size of nine individuals for each experiment.
